# Peripheral versus central mechanisms of the cannabinoid type 2 receptor agonist AM1710 in a mouse model of neuropathic pain

**DOI:** 10.1002/brb3.1850

**Published:** 2020-09-25

**Authors:** Jenny L. Wilkerson, Lauren B. Alberti, Audra A. Kerwin, Catherine A. Ledent, Ganesh A. Thakur, Alexandros Makriyannis, Erin D. Milligan

**Affiliations:** ^1^ Department of Neurosciences Health Sciences Center School of Medicine University of New Mexico Albuquerque NM USA; ^2^ Universite libre de Bruxelles Brussels Belgium; ^3^ Center for Drug Discovery Northeastern University Boston MA USA; ^4^ Department of Pharmacodynamics College of Pharmacy University of Florida Gainesville FL USA

**Keywords:** cannabinoid, MCP‐1/CCL‐2, mouse, paraffin immunohistochemistry, spectral analysis

## Abstract

The CB_2_R agonist AM1710, examined in animal models of peripheral neuropathy, is effective in controlling aberrant light touch sensitivity, referred to as mechanical allodynia. However, nonspecific binding of AM1710 to CB_1_R, either peripherally or centrally, could be partially responsible for the analgesic effects of AM1710. Thus, we sought to determine in mice whether spinal (intrathecal; i.t.) or peripheral AM1710 administration could lead to anti‐allodynia by reducing the protein expression of spinal and dorsal root ganglia (DRG) proinflammatory cytokines and elevating the anti‐inflammatory cytokine interleukin‐10 (IL‐10) in the absence of CB_1_R. Macrophage cell cultures were examined to characterize AM1710‐mediated suppression of the proinflammatory cytokine tumor necrosis factor‐alpha (TNF‐α). Either i.p. or i.t. AM1710 reversed CCI‐induced mechanical allodynia to sham levels in CB_1_R (−/−), (+/−), (+/+) mice. CCI‐induced neuropathy decreased IL‐10 immunoreactivity (IR) in the dorsal root ganglia (DRG) and the dorsal horn of the spinal cord, with i.t. AM1710 restoring basal IL‐10 IR. CCI‐induced elevations in proinflammatory cytokine IR were decreased within the spinal cord only after i.t. AM1710 in all mouse genotypes. Meanwhile, within DRG tissue from neuropathic mice, proinflammatory cytokines were decreased following either i.p. or i.t. AM1710. Analysis of cultured supernatants revealed AM1710 decreased TNF‐alpha protein. We conclude that CB_1_R is dispensable for either peripheral or central anti‐allodynic actions of AM1710 in neuropathic mice. Cannabinoid CB_2_R agonists produce heightened spinal IL‐10 which may be clinically relevant to successfully treat neuropathic pain.

## Graphical abstract

Either i.p. or i.t. AM1710 reversed CCI‐induced mechanical allodynia in CB1R (−/−), (+/−), (+/+) mice similar to sham control threshold levels. The absence of the CB1R following CCI‐induced neuropathy revealed greater significant increases in IL‐10 immunoreactivity (IR) in the dorsal root ganglia (DRG) and the dorsal horn of the spinal cord in (−/−) and (+/−) mice that was further elevated following i.t. and i.p. AM1710. The absence of CB1R may allow for enhanced tissue/axon damage‐associated signaling to the DRG and spinal cord, resulting in endogenous compensatory increases in IL‐10 expression.

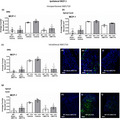

## 1 INTRODUCTION

The dorsal horn of the spinal cord and dorsal root ganglia (DRG) are well characterized to house neurons critical for processing information underlying neuropathic pain. It has been found that glia (i.e., astrocytes, microglia, and DRG satellite cells) mediate chronic pathological pain signaling (Abbadie et al., 2009; De Leo, Tawfik, & LaCroix‐Fralish, 2006; Haight, Forman, Cordonnier, James, & Tawfik, 2019; Ji, Chamessian, & Zhang, 2016; Milligan et al., 2003). Under healthy conditions, constitutive glial activation is correlated with immune surveillance and the maintenance of homeostasis. However, continuous glial activation can lead to aberrant, pathological signaling with the release of proinflammatory cytokines. These proinflammatory cytokines include both tumor necrosis factor‐alpha (TNF‐α) and interleukin (IL)‐1β. Proinflammatory cytokines are known to be critical components of chronic neuropathic pain (Inoue & Tsuda, 2018; Milligan & Watkins, 2009; Vanderwall & Milligan, 2019). The chemokine CCL2 (cystine–cystine motif ligand 2), also known as monocyte chemo‐attractant protein‐1 (MCP‐1) (Deshmane, Kremlev, Amini, & Sawaya, 2009), exists as a neuron‐to‐glia signaling molecule. MCP‐1 is released from spinal cord presynaptic neurons found within dorsal horn superficial lamina as well as peripheral nociceptors, binds to and activates its receptor CCR2 expressed on immune cells such as macrophage and other myeloid lineage cells, and leads to cytokine release that acts on their receptors expressed on nearby glia as well as neurons resulting in pathological pain (Abdallah et al., 1996; Hu, Zheng, Yang, Wang, & Ji, 2012; Hu & McLachlan, 2002; Jeon, Lee, & Cho, 2009). MCP‐1 is well characterized to be upregulated in spinal and DRG neuronal cell bodies in numerous animal models of neuropathic pain (Hu et al., 2012; Echeverry, Shi, Rivest, & Zhang, 2011; Jeon et al., 2009; Dansereau et al., 2008; Jung, Toth, White, & Miller, 2008; Bhangoo et al., 2007; Morin et al., 2007; Zhang et al., 2007; 2006; White et al., 2005) as well as in astrocytes (Gao et al., 2009). Acting in an opposing fashion to the pronociceptive effects of IL‐1β and TNF‐α, it has been established that the anti‐inflammatory actions of the cytokine IL‐10 reverse neuropathic pain in several animal models (Vanderwall & Milligan, 2019; Ledeboer et al., 2007; Milligan et al., 2006; Soderquist et al., 2010). Although anatomically distinct, the relative contribution of microglial spinal cord cytokines compared to cytokines from the DRG in the ongoing maintenance of chronic pain conditions is not well understood.

The cannabinoid 1 receptor (CB_1_R) is predominately found on peripheral nociceptive cell bodies within the DRG and on pre‐ and postsynaptic neurons in the superficial lamina of the dorsal horn, including GABA‐ergic interneurons (Donvito et al., 2018; Salio, Fischer, Franzoni, & Conrath, 2002). The cannabinoid 2 receptor (CB_2_R) is predominately found on peripheral innate immune cells and on spinal cord microglia. Activation of either CB_1_R or CB_2_R produces analgesia (Ahmed et al., 2010; Beltramo, 2009; Curto‐Reyes, Llames, Hidalgo, Menendez, & Baamonde, 2010; Donvito et al., 2018; Maresz et al., 2007; Romero‐Sandoval & Eisenach, 2007; Romero‐Sandoval, Nutile‐McMenemy, & DeLeo, 2008). However, actions of agonists at the CB_1_R produce a number of unwanted physiological side effects other than pain control, which diminishes their potential clinical application. Alternatively, activation of the CB_2_R does not induce CB_1_R‐related physiological side effects. The CB_2_R agonist AM1710 is from the cannabilactone classification (Khanolkar et al., 2007; Rahn et al., 2011), and we previously reported that AM1710 prevents and reverses allodynia, light touch mechanical sensitivity in rats. Allodynia is a significant and frequent manifestation of neuropathic pain (Wilkerson et al., 2012a). In correlation with behavioral reversal, we found a distinct anti‐inflammatory effect after intrathecal administration of AM1710 in both the dorsal horn of the spinal cord and corresponding DRG. However, CB_1_Rs may be greater in number than CB_2_Rs in the spinal cord and periphery, and consequently, may play a much greater role in pain control. Immunohistochemical staining for the CB_2_R has been problematic leading to difficulty in quantifying numbers of CB_1_R versus CB_2_R under normal and pathological conditions. Therefore, while AM1710 has a ~54‐fold greater affinity for the CB_2_R over the CB_1_R (Khanolkar et al., 2007), nonspecific actions at the CB_1_R as a major factor contributing to the anti‐allodynic effects of either peripherally or centrally administered AM1710 cannot be ruled out.

Our previous study in rats demonstrated that under neuropathic pain conditions, AM1710 increased DRG and spinal expression of IL‐10, decreased the expression of IL‐1β as well as phospohorylated‐p38MAPK (a necessary kinase for TNF‐α production), and, in the same report, identified a reduction in TNF‐α protein content in DRG as measured by ELISA (Wilkerson et al., 2012a). However, due to the redundancy of immune cytokines, it was not clear whether AM1710 could lead to direct changes in TNF‐α. Thus, conducting a discrete in vitro cell experiment focused on the effect of AM1710 in reducing TNF‐α protein levels could serve as complementary to the existing data. These results could further support AM1710 exerts anti‐inflammatory actions via the IL‐10 and MAPK pathways.

The goal of the studies in the current report was to examine whether AM1710 (1) controls allodynia produced by unilateral sciatic nerve damage, (2) concurrently alters lumbar spinal cord and/or lumbar DRG expression of IL‐1β, IL‐10, and/or MCP‐1 through CB_2_R‐specific actions, and (3) whether AM1710 is capable of suppressing TNF‐α release from cultured macrophage, cells which are characterized to infiltrate neural tissue under neuropathic conditions contributing to the pool of pronociceptive cytokines (Chen, Donnelly, & Ji, 2019; Kiguchi, Kobayashi, Saika, Matsuzaki, & Kishioka, 2017). Gene‐deleted or reduced CB_1_R (i.e., CB_1_R (−/−) and CB_1_R (+/−) mice) were employed to examine the CB_2_R specificity of AM1710. Following immunohistochemical procedures to detect IL‐1β, IL‐10, and MCP‐1 expression, immunofluorescent quantification of stained sections of the lumbar dorsal horn and lumbar DRG was analyzed using Nuance^TM^ computer‐assisted Multispectral Imaging System. The data confirm CB_1_Rs are dispensable for the anti‐allodynic actions of AM1710 given peripherally or intrathecally. Additionally, AM1710 decreased MCP‐1, a key chemokine involved in generating neuropathic pain.

## 2 METHODS

### 2.1 Animals

A total of 173 pathogen‐free adult male mice (24–39 g) from a CD1 genetic background were used in all experiments. The weight range in the mice correlated with the age range (1–3 months) as well as the CB_1_R genotype differences. The mice with a genetic deletion of the CB_1_R had a tendency to weigh less than age‐matched controls, but this did not lead to observable differences in withdrawal responses during behavioral testing. Mice were housed in a thermal and light‐controlled (12‐hr light/dark; lights on at 6:00 a.m.) room, with ad libitum access to standard rodent chow and water. All procedures were approved by the Institutional Animal Care and Use Committee (IACUC) at the University of New Mexico Health Sciences Center and were in a manner conforming to the National Institutes of Health Guide for the Care and Use of Laboratory Animals (National Research Council, 2011). These studies are reported in a manner conforming to the ARRIVE guidelines for studies involving animals (Kilkenny, Browne, Cuthill, Emerson, & Altman, 2010; McGrath, Drummond, McLachlan, Kilkenny, & Wainwright, 2010).

Heterozygous CB_1_R (+/−) mice with a CD1 background were generated and were a generous gift from Dr. C. Ledent (Ledent et al., 1999). Of note, it has been established that heterozygous CB_1_R (+/−) mice do not display typical behavioral endpoints observed with CB_1_R agonist administration. Heterozygous CB_1_R (+/−) and knockout CB_1_R (−/−) mice, along with their wild‐type CB_1_R (+/+) littermates, were used in all experiments.

The following primer sequences were used in PCR genotyping of these mice:

**Table:** 

GGG TGA GGA GAC ATG CCT GGT GA	CB_1_R (+/+) wild‐type forward primer
AGA GGT GCC AGG AGG GAA CCC TA	CB_1_R (+/+) wild‐type reverse primer
CCT TGC GCA GCT GTG CTC GA	CB_1_R (−/−) knockout forward primer
GAA CAG TTC GGC TGG CGC GA	CB_1_R (−/−) knockout reverse primer

### 2.2 Drugs

The CB_2_R agonist, 3–1(1’,1’‐dimethylheptyl)‐1‐hydroxy‐9‐methoxy‐6H‐benzo[c]‐chromene‐6‐one (AM1710) (Khanolkar et al., 2007; Wilkerson et al., 2012a) was used in these experiments. As previously reported, AM1710 was dissolved in 100% ethanol and then diluted in sterile water (Hospira Inc). The final concentration of AM1710 was 1 mg/mL containing 5% ethanol. Also as described previously, sterile water containing 5% ethanol was used as vehicle for all compounds (Wilkerson et al., 2012a). The selective CB_2_R antagonist (3 μg/3 μl), 6‐Iodo‐2‐methyl‐1‐[2‐(4‐morpholinyl)ethyl]‐1H‐indol‐3‐yl](4‐methoxyphenyl)methanone, (AM630) was purchased (cat # 1,120; Tocris Bioscience). As described for AM1710, AM630 was dissolved in 100% ethanol and then diluted in sterile water (Hospira Inc). The final concentration of AM630 was 1 mg/ml containing 5% ethanol. Pilot experiments determined i.t. administration of 5 μg AM1710 produced maximal anti‐allodynia by 1.5 hr, with anti‐allodynia no longer present by 5 hr after drug administration (data not shown).

### 2.3 Behavioral assessment of allodynia

Baseline (BL) responses to the von Frey assay were assessed after animals were habituated to the testing area, with testing conducted with modifications from that previously described (Ignatowska‐Jankowska et al., 2015). Briefly, mice were put on 2‐mm‐thick parallel bars, with wire mesh spaces 1 mm apart. Mice were habituated for approximately 30 min for 4 consecutive days. Mice were not restrained and were individually placed under an inverted wire mesh cup. Behavioral testing was conducted in a sound‐, light‐, and temperature‐controlled room during the first half of the light cycle. The von Frey assay uses a range of calibrated monofilaments (2.83–4.31 log stimulus intensity; North Coast Medical) applied to the surface of the hindpaw for a maximum 3 s, as previously described (Vanderwall et al., 2018). The total responses at each tested monofilament were used in the calculation of the absolute (50%) paw withdrawal threshold via the computer program PsychoFit (http://psych.colorado.edu/~lharvey; RRID:SCR_015381), as previously described (Dengler et al., 2013; Milligan et al., 2000). This software fits a Gaussian integral psychometric function to the withdrawal responses for each of the monofilaments tested via a maximum‐likelihood fitting method (Milligan et al., 2000). The log threshold values obtained from the PsychoFit program were then used for subsequent statistical analyses. All behavioral testing was conducted within the first four hours of the light cycle to decrease variance associated with circadian rhythm differences. All behavioral testing was performed in a blinded fashion by J.L.W.

### 2.4 Chronic constriction injury (CCI) surgery

After BL testing was completed, chronic constriction injury (CCI) of the sciatic nerve surgery was performed as previously described (Ignatowska‐Jankowska et al., 2015). In isoflurane (induction 5% vol. followed by 2.0% in oxygen)‐anesthetized mice, the middle and lower back, as well as the left thigh, was shaved, washed with diluted Bacti‐Stat AE (EcoLab Health Care Division), cleansed with water, and finally daubed with 70% EtOH. The sciatic nerve was isolated and loosely ligated with 3 segments of 5–0 chromic gut sutures (Ethicon) via aseptic techniques. Apart from the loose nerve ligation, the sham surgical procedure was identical to CCI surgical procedure. The thigh muscle was secured using a single 4–0 sterile silk suture (Ethicon). Mice recovered from the effects of isoflurane within approximately 5 min. Surgical group placement was randomly assigned.

### 2.5 Acute intrathecal (i.t.) injection

For experiments requiring intrathecal injections, experimental compounds were delivered via an acute i.t. catheter. An injection catheter was crafted in a manner as previously described (Beutler, Banck, Walsh, & Milligan, 2005; Milligan, Langer, et al., 2005; Milligan, Sloane, et al., 2005; Milligan et al., 2006). Briefly, a 27‐gauge needle without the plastic hub was placed into one end of polyethylene (PE)‐20 tubing. At the other end of the PE‐20, tubing was placed a needle segment of a different 27‐gauge needle with the hub connected to a 10‐μl Hamilton syringe. Using prior publications as a guideline, an i.t. dose of 5 μg AM1710 (Wilkerson et al., 2012a) or 3 μg of the selective CB_2_R antagonist, AM630 (Gu et al., 2011), was used. Additionally, prior work has demonstrated that i.t. AM630 given alone to wild‐type mice does not alter normal hindpaw sensory thresholds compared to vehicle control mice, and as such, *an analysis of i.t. AM630 in wild‐type mice in the current report was omitted* (Deng et al., 2015). Drug or equivolume vehicle was loaded in preparation for injection, and the 27‐gauge needle was placed into the dorsal aspect of the L5‐L6 vertebrae. Upon needle insertion, a slight leg and/or tail twitch was typically observed indicating correct insertion. Injections occurred across a 5‐s duration. Mice were randomly assigned to drug or vehicle treatment groups. Upon removal from anesthesia (~5 min), all mice were observed to recover normal motor function.

### 2.6 Acute intraperitoneal (i.p.) injection

An acute i.p. injection of either AM1710 or vehicle was used, in CCI‐operated or sham‐operated mice. Prior reports (Rahn et al., 2011) indicate a maximally effective dose of 25 mg kg^−1^ ml^−1^ (5 mg/kg) AM1710, which was utilized in these studies.

### 2.7 Immunohistochemical procedures from CCI‐treated mice

After behavioral testing, tissues were collected and processed for immunohistochemistry as previously described (Curry et al., 2018; Wilkerson et al., 2012a, 2012b). In brief, mice were given an overdose of i.p. sodium phenobarbital (Sleepaway, Fort Dodge Animal Health), followed by transcardial saline and 4% paraformaldehyde perfusion. Vertebral columns containing intact spinal cords were collected, placed in 4% paraformaldehyde at 4°C overnight, then subsequently decalcified, and paraffin‐embedded. Adjacent 7‐μm‐thick tissue sections were placed on Superfrost Plus slides (VWR) and then underwent deparaffinization, rehydration, and antigen retrieval procedures as previously described (Curry et al., 2018; Wilkerson et al., 2012a, 2012b).

Tissues were then incubated with 5% normal donkey serum (NDS), in PBS (pH 7.4) for 2 hr, followed by primary antibody incubation overnight. All antibody methods were conducted as previously described (Curry et al., 2018; Wilkerson et al., 2012a, 2012b). For the detection of GFAP (astrocytes), a rabbit anti‐GFAP polyclonal antibody was used (1:1,000; AB5804, RRID:AB_2109645; Millipore‐Sigma). For the detection of Iba‐1(microglia), a rabbit polyclonal anti‐Iba‐1 antibody was used (1:300; 019–19741, RRID:AB_839506; Wako). For the detection of IL‐10, a goat polyclonal anti‐IL10 antibody was used (1:250; AF519, RRID:AB_355408; R&D Systems). For the detection of IL‐1β, a rat monoclonal anti‐mouse IL‐1β antibody was used (1:300; 74,134, RRID:AB_1123317; Santa Cruz Biotechnology). For the detection of MCP‐1/CCL2, an Armenian hamster anti‐rat monoclonal antibody was used (1:100; clone 2H5, cat. # NB100‐78196, RRID:AB_1083117; Novus Biologicals). For IL‐10 staining, tissues were incubated with biotinylated secondary antibody for 1 hr, developed with the VECTASTAIN ABC Elite Kit (Vector Labs) and TSA Plus Fluorescein System (PerkinElmer Life Sciences). All other slides underwent secondary antibody incubation for 2 hr. Slides were then rinsed in PBS and cover‐slipped with VECTASHIELD containing the nuclear stain 4’,6‐diamidino‐2‐phenylindole (DAPI) (Vector Labs).

### 2.8 Immunofluorescent spectral image analysis

For these studies, images of the spinal cord and DRGs were captured by a Nikon inverted fluorescent microscope, at 20x magnification, with a Nuance Spectral Camera (Cambridge Research & Instrumentation), as previously described (Wilkerson et al., 2012a, 2012b). Briefly, utilizing the Nuance computer software, the fluorescent wavelength emission spectra range was initially determined for each fluorophore by using a control slide containing only the fluorophore of interest. The protein marker intensity threshold was determined and was consistently applied throughout all image analysis. After image analysis, software conversion allowed for the fluorescent wavelength intensities for each fluorophore to be converted to numerical values.

From this analysis, we report the average count of fluorescent emission intensity per second exposure, per mm^2^ (i.e., fluorescent intensity average count/second/mm^2^). This measurement encompasses both the density and the intensity of the fluorophore examined. In this manner, four tissue sections per mouse (*N* = 3) were randomly selected and analyzed.

### 2.9 RAW264.7 cell culture, AM1710 incubation, and lipopolysaccharide stimulation

Mouse macrophage‐like RAW 264.7 cells were obtained from American Type Culture Collection (cat# TIB‐71; ATCC Manassas) and cultured in Dulbecco's modified Eagle's medium (Sigma‐Aldrich) supplemented with 10% heat‐inactivated fetal bovine serum and 100 U/ml penicillin and 100 μg/ml streptomycin (Gibco–Life Technologies) at 37°C under humidified 5% CO2 atmosphere. Cells were seeded at 150,000 cells/mL in 24‐well plates and grown overnight. The following day, cells were treated with vehicle, 10 pg/mL, 1 ng/ml, 10 ng/ml, and 100 ng/ml AM1710 for 4 hr after a 10‐min exposure to the gram‐negative cell wall protein, lipopolysaccharide (LPS) at a concentration of 500 ng/ml (E. coli #055:B5; Sigma‐Aldrich). Cells were washed with fresh media after treatments and incubated for 24 hr, followed by collection of supernatants 24 hr after treatment. All treatments were conducted in quadruplicates, and extracellular proteins (supernatants) or total cell lysates were used for protein as outlined below.

### 2.10 Cell culture protein quantification by ELISA

RAW 264.7‐treated cell supernatants (50 µl) or 10 ug total cellular protein in quadruplicates were collected separately into vials and frozen in liquid nitrogen. Vials were stored at −80*C and then assayed for extracellular or intracellular protein quantification. Using standardized instructions from the manufacturer, ELISAs were performed for IL‐10 and TNF‐α (R&D Systems).

### 2.11 Data analysis

Psychometric behavioral analysis was performed as described above and previously detailed (Treutwein & Strasburger, 1999; Wilkerson et al., 2012a, 2012b). For statistical analysis of behavior, a 1‐way ANOVA was used at BL, and a 2‐way repeated‐measures ANOVA was used after surgery. All other data analysis was performed using a one‐way ANOVA. A *p*‐value of <.05 was considered statistically significant (Wilkerson et al., 2020). GraphPad Prism version 4.03 (GraphPad Software Inc.) was utilized for all statistical analyses, and Bonferroni's test was utilized as the post hoc assessment. Data are expressed as mean ± *SEM*.

## 3 RESULTS

### 3.1 CB_1_R (−/−) mice display similar allodynia profiles to CB_1_R (+/−) and CB_1_R (+/+) littermates

**Figure 1:**
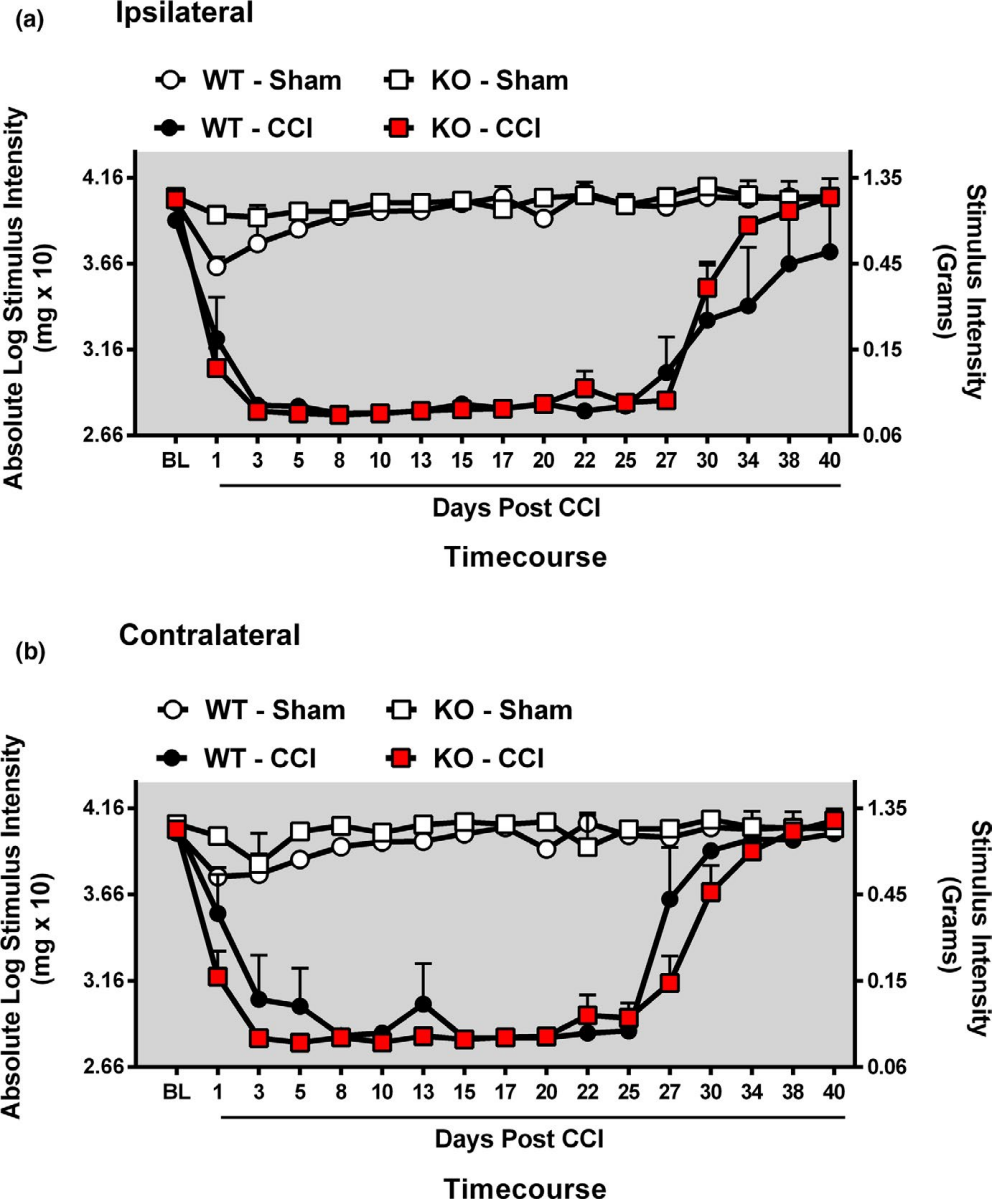
Characterization of length and severity of bilateral allodynia in CB_1_R (−/−) and (+/+) mice. Characterization of length and severity of bilateral allodynia in CB_1_R (−/−) KO and (+/+) WT mice. (a, b) Mice developed and maintained CCI‐induced allodynia regardless of functional CB_1_R copy number. Before surgical manipulation, all experimental groups exhibited similar ipsilateral and contralateral BL thresholds. CCI surgery produced significant bilateral allodynia at day 5 post‐CCI through day 27 post‐CCI compared to sham‐treated animals. Data reflect mean ± *SEM*, *n* = 7 mice per group for all groups except *n* = 6 within the KO‐Sham group

Functional knockout of CB_1_R produced no overt changes in the bilateral allodynia profiles obtained with CCI. Before surgery, all groups displayed similar bilateral (ipsilateral and contralateral) BL responses (ANOVA, *F*_(5,40)_ = 1.661; *p* = .1693 ANOVA, *F*_(5,40)_ = 1.659; *p* = .1697, respectively) (Figure 1a,b, Figure S1a,b). Bilateral allodynia developed regardless of the CB_1_R genetic profile by day 5 after CCI surgery compared to sham‐operated mice (ANOVA, *F*_(17,72)_ = 9.755; *p* < .0001 ANOVA, *F*_(17,72)_ = 20.09; *p* < .0001, respectively, for ipsilateral and contralateral allodynia). Bilateral allodynia was present until day 27 postsurgical manipulation, after which allodynia spontaneously resolved (ANOVA, *F*_(77,395)_ = 2.956; *p* < .0001 ANOVA, *F*_(77,395)_ = 5.483; *p* < .0001, respectively). All groups of sham‐operated mice exhibited thresholds similar to baseline responses.

### 3.2 Intrathecal AM1710 alters behavior and protein expression in CB_1_R (−/−) mice

**Figure 2:**
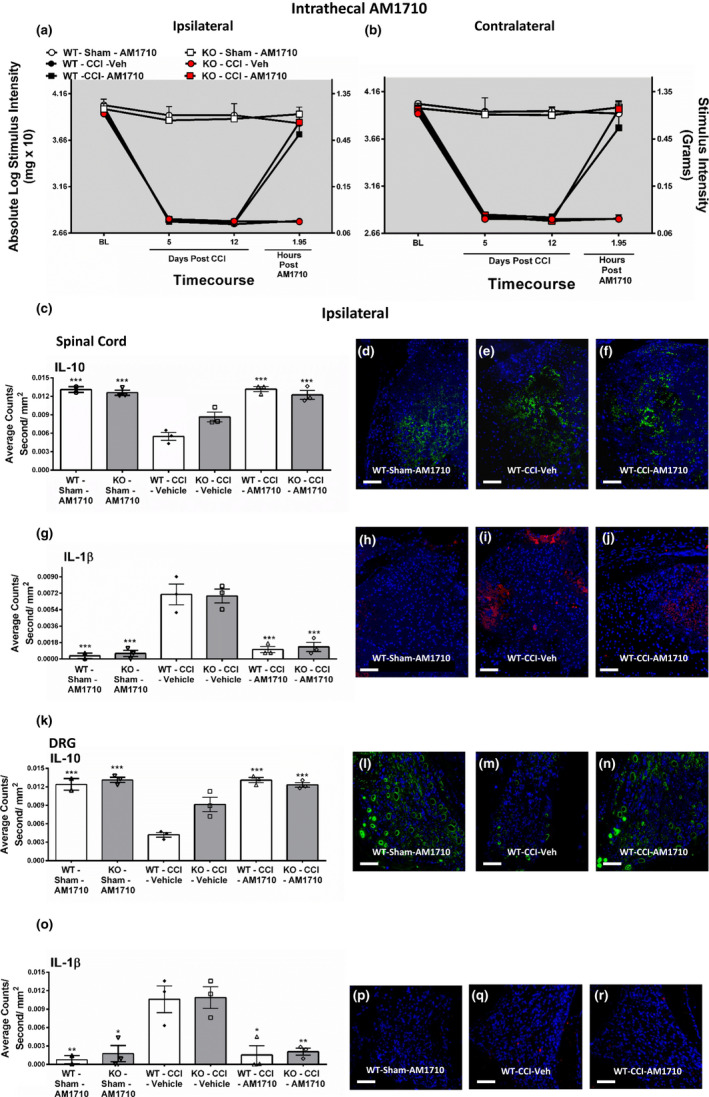
Intrathecal AM1710, a cannabinoid 2 receptor agonist, reverses CCI‐induced allodynia and modulates cytokines. Intrathecal AM1710 reversed CCI‐induced allodynia and modulated cytokines independent of CB_1_R. (a, b) AM1710 reversed CCI‐induced allodynia independent of CB_1_R actions. Before surgical manipulation, all experimental groups exhibited similar ipsilateral and contralateral BL thresholds and CCI surgery produced significant bilateral allodynia at days 5 and 12 following injury compared to sham‐treated animals. Responses from AM1710 (5 μg) maximally reversed CCI‐induced allodynia at ~2 hr after intrathecal administration. From these mice, ipsilateral immunofluorescent intensity quantification from 7‐µm‐thick sections of dorsal horn spinal cord and corresponding DRG were performed. (c) IL‐10 expression in dorsal horn spinal cord was decreased in CCI‐treated mice that received i.t. vehicle compared to control sham‐treated mice given AM1710, while IL‐10 IR recovered to sham levels in CCI neuropathic mice given i.t. AM1710. (d, e, f) Representative spectrally unmixed images at 20x magnification of IL‐10 fluorescent labeling (green) with DAPI nuclear stain (blue) in dorsal horn spinal cord. (g) Compared to sham controls, IL‐1β expression was increased in the dorsal horn spinal cord of CCI‐treated animals given i.t. vehicle of AM1710. However, i.t. AM1710 in CCI‐treated mice robustly suppressed increases in IL‐1β IR. (h, i, j) Representative spectrally unmixed images at 20x magnification of IL‐1β fluorescent labeling (red) with DAPI nuclear stain (blue) in dorsal horn spinal cord. (k) IL‐10 expression in DRG was decreased in CCI‐treated mice that received i.t. vehicle compared to control sham‐treated mice given AM1710, while IL‐10 IR recovered to sham levels in CCI neuropathic mice given i.t. AM1710. (l, m, n) Representative spectrally unmixed images at 20x magnification of IL‐10 fluorescent labeling (green) with DAPI nuclear stain (blue) in DRG. (o) Compared to sham controls, IL‐1β expression was increased in the DRG of CCI‐treated animals given i.t. vehicle of AM1710. However, i.t. AM1710 in CCI‐treated mice robustly suppressed increases in IL‐1β IR. (p, q, r) Representative spectrally unmixed images at 20x magnification of IL‐1β fluorescent labeling (red) with DAPI nuclear stain (blue) in DRG. In all images, the scale bar is equal to 50 µm. ***p < .0001, **p < .001, *p < .05 versus. WT‐CCI‐Vehicle. Data reflect mean ± *SEM*. For behavioral data, *n* = 6 mice per group for all groups except *n* = 5 within the KO‐Sham‐AM1710 group. For immunohistochemistry data, *n* = 3 mice per group for all groups except *n* = 2 within the WT‐Sham‐AM1710 group

Before surgery, all mice displayed similar bilateral (ipsilateral and contralateral) BL responses (ANOVA, *F*_(8,52)_ = 1.199; *p* = .3214 ANOVA, *F*_(8,52)_ = 1.172; *p* = .3369, respectively, for ipsilateral and contralateral thresholds) (Figure 2a,b, Figure S2a,b). After surgery, bilateral allodynia was present in CCI‐operated mice at day 5 and day 12 compared to sham‐operated mice (ANOVA, *F*_(15,89)_ = 137.4; *p* < .0.0001 ANOVA, *F*_(15,89)_ = 207.3; *p* < .0001, respectively). Throughout the time course of the experiment, 5 µg i.t. AM1710 injection in sham‐operated mice did not influence light touch threshold responses. However, i.t. AM1710 fully reversed CCI‐induced allodynia, observed at 2 hr after injection in CB_1_R (+/+) wild‐type (WT), (+/−) heterozygous (Het), or (−/−) homozygous knockout (KO) mice.

Bilateral immunoreactivity (IR) for IL‐10 was dramatically decreased in both the ipsilateral and contralateral dorsal horn of the spinal cord taken from CCI mice with i.t. vehicle when compared to spinal cords taken from sham mice with i.t. AM1710 (Figure 2c, Figure S2c,d). However, in CCI‐operated mice AM1710 further increased bilateral IL‐10 IR, with levels similar to those observed in sham‐operated controls (ANOVA, *F*_(8,24)_ = 14.91; *p* < .0001 ANOVA, *F*_(8,24)_ = 5.071; *p* = .0028, respectively). Representative fluorescent images corresponding to data examined by microscopic analysis are shown of ipsilateral dorsal horn of the spinal cord from wild‐type mice with either sham surgery with i.t. AM1710 (Figure 2d) or CCI surgery with i.t. vehicle (Figure 2e) or with AM1710 (Figure 2f) corresponding to the quantitative IL‐10 IR data are shown.

Regardless of genotype, CCI produced a bilateral increase in IL‐1β IR within the dorsal horn of the spinal cord in vehicle‐treated animals (Figure 2g, Figure S2e,f). Meanwhile, in CCI‐operated mice i.t. AM1710 completely suppressed the elevated IL‐1β IR in the dorsal horn (ANOVA, *F*_(8,24)_ = 19.34; *p* < .0001 ANOVA, *F*_(8,24)_ = 39.90; *p* < .0001, respectively). Representative IL‐1β IR fluorescent images corresponding to data examined by microscopic analysis are shown of sham mice with i.t. AM1710 (Figure 2h), CCI mice with i.t. vehicle (Figure 2i), or CCI mice with i.t. AM1710 (Figure 2j).

Similar to observations of the dorsal horn of the spinal cord, bilateral IL‐10 IR in ipsilateral and contralateral DRG taken from CCI neuropathic mice was dramatically decreased compared to IL‐10 IR observed in sham‐operated mice regardless of genotype (Figure 2k, Figure S2g,h). However, in CCI‐operated mice i.t. AM1710 significantly increased IL‐10 IR bilaterally in the DRGs to basal levels observed in sham‐operated mice (ANOVA, *F*_(8,24)_ = 13.52; *p* < .0001 ANOVA, *F*_(8,24)_ = 6.045; *p* = .0011, respectively). Representative fluorescence images corresponding to data examined by microscopic analysis of ipsilateral DRG from wild‐type mice with either sham treatment with i.t. AM1710 (Figure 2l) or CCI treatment with i.t. vehicle (Figure 2m) or with AM1710 (Figure 2n).

A striking pattern of bilateral increases in IL‐1β IR in the DRG was observed in CCI mice compared to sham‐operated mice (Figure 2o, Figure S2i,j). However, in CCI‐operated mice AM1710 decreased IL‐1β IR in DRGs in CCI mice (ANOVA, *F*_(8,24)_ = 19.34; *p* < .0001 ANOVA, *F*_(8,24)_ = 39.90; *p* < .0001, respectively). Representative fluorescence images of ipsilateral DRG from CB_1_R (+/+) sham mice with i.t. AM1710 (Figure 2p) and CCI mice with i.t. vehicle (Figure 2q) or AM1710 (Figure 2r) corresponding to the quantitative data are shown.

### 3.3 Pretreatment with CB_2_R antagonist blocks intrathecal AM1710 actions

**Figure 3:**
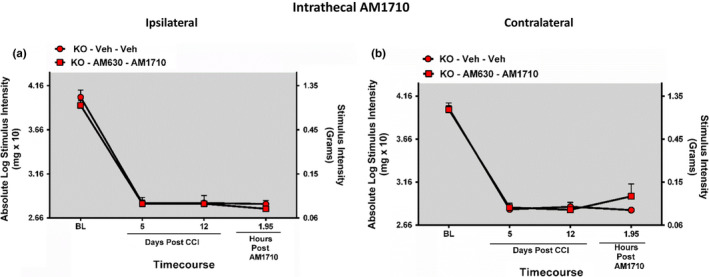
Intrathecal AM1710 anti‐allodynia efficacy requires CB_2_R actions. (a, b) Intrathecal pretreatment with the CB_2_R selective antagonist AM630 30 min before i.t. administration of AM1710 blocks the anti‐allodynia effects of AM1710 in CB_1_R KO mice. Data reflect mean ± *SEM*, *n* = 6 mice per group

Neuropathic mice lacking functional CB_1_Rs and pretreated with the selective CB_2_R antagonist AM630 thirty minutes prior i.t. AM1710 did not exhibit reversal from allodynia (ANOVA, *F*_(3,19)_ = 0.9822; *p* = .3918 ANOVA, *F*_(3,19)_ = 1.729; *p* = .2029, respectively) (Figure 3a,b).

### 3.4 Intraperitoneal AM1710 reverses CCI‐altered allodynia

In pilot studies, we examined an AM1710 i.p. dose of 25 mg kg^−1^ ml^−1^ based on prior reports (Rahn et al., 2011). This dose of AM1710 was sufficient to reverse bilateral allodynia, with maximal reversal occurring at 0.5 hr, which continued to 1 hr after administration, with anti‐allodynic effects no longer present by 2 hr (data not shown). Based on these observations, behavioral hindpaw assessment for allodynia and tissue collection occurred at ~0.5 hr.

**Figure 4:**
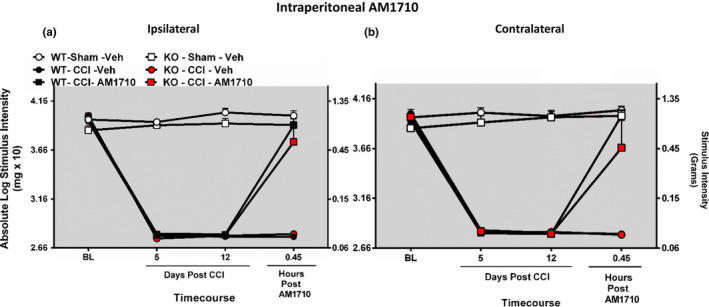
Intraperitoneal AM1710 reverses CCI‐induced allodynia in a CB_1_R independent manner. Intraperitoneal AM1710, a cannabinoid 2 receptor agonist, reverses CCI‐induced allodynia in a CB_1_R‐independent manner. (a, b) AM1710 reverses CCI‐induced allodynia independent of CB_1_R actions. Before surgical manipulation, all experimental groups exhibited similar ipsilateral and contralateral BL thresholds and CCI surgery produced significant bilateral allodynia at days 5 and 12 following injury compared to sham‐treated animals. Responses from AM1710 (25 mg kg^‐1^ ml^‐1^) maximally reversed CCI‐induced allodynia at ~30 min after intraperitoneal administration. Data reflect mean ± *SEM*, *n* = 6 mice per group for all groups except *n* = 5 within the KO‐Sham‐AM1710 group

In separate groups of mice, prior to surgical manipulation, all mice displayed similar bilateral (ipsilateral and contralateral) BL behavioral thresholds (ANOVA, *F*_(8,52)_ = 2.085; *p* = .0574 ANOVA, *F*_(8,52)_ = 1.341; *p* = .2486, respectively) (Figure 4a,b, Figure S3a,b). Replicating our prior experiment, bilateral allodynia was present at day 5 and day 12 in all CCI‐operated mice compared to sham‐operated mice (ANOVA, *F*_(15,89)_ = 182.4; *p* < .0001 ANOVA, *F*_(15,89)_ = 143.5; *p* < .0001, respectively). On Day 12, following i.p. vehicle, no change in sensory threshold responses was observed in sham‐operated mice. Meanwhile, i.p. AM1710 produced reversal from allodynia in mice with CCI, regardless of genotype.

### 3.5 Intraperitoneal AM1710 alters both dorsal horn and DRG protein expression

**Figure 5:**
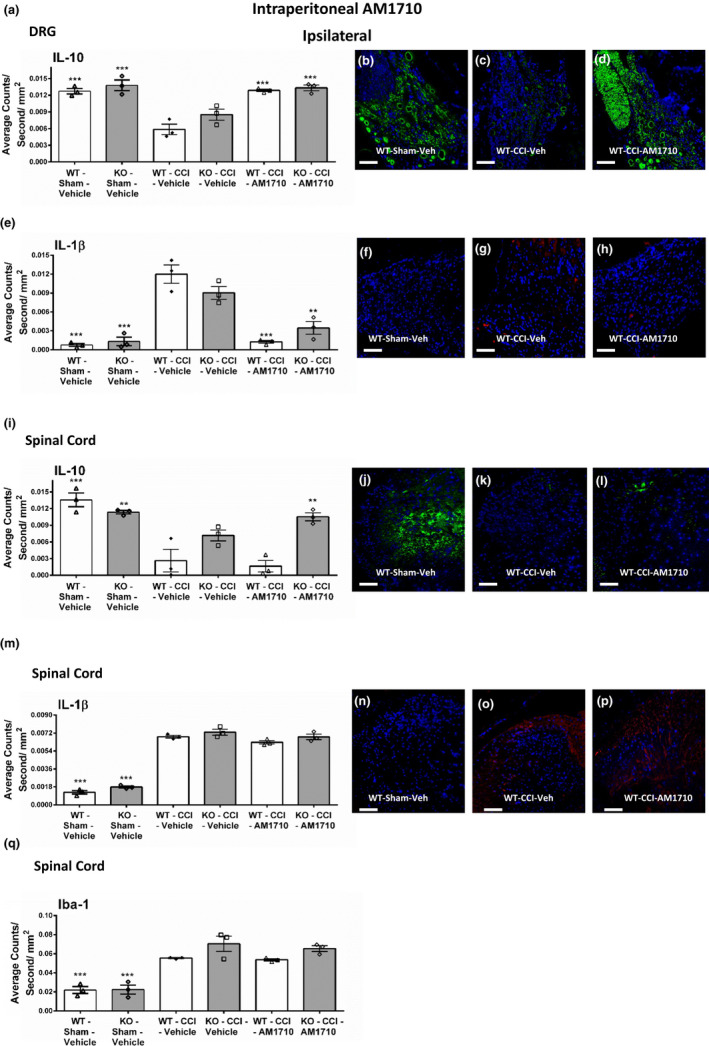
Ipsilateral immunofluorescent intensity quantification of tissues following intraperitoneal AM1710. Ipsilateral immunofluorescent intensity quantification from 7‐µm‐thick sections of DRG and dorsal horn spinal cord from behaviorally verified CB_1_R KO, Het, WT mice, following i.p. vehicle or AM1710. (a) IL‐10 expression in DRG was decreased in CCI‐treated mice that received i.p. vehicle compared to control sham‐treated mice given AM1710, while IL‐10 IR recovered to sham levels in CCI neuropathic mice given i.p. AM1710. (b, c, d) Representative spectrally unmixed images at 20x magnification of IL‐10 fluorescent labeling (green) with DAPI nuclear stain (blue) in DRG. (e) Compared to sham controls, IL‐1β expression was increased in the DRG of CCI‐treated animals given i.p. vehicle of AM1710. However, i.p. AM1710 in CCI‐treated mice robustly suppressed increases in IL‐1β IR. (f, g, h) Representative spectrally unmixed images at 20x magnification of IL‐1β fluorescent labeling (red) with DAPI nuclear stain (blue) in DRG. (i) IL‐10 expression in dorsal horn spinal cord was decreased in CCI‐treated mice that received i.p. vehicle compared to control sham‐treated mice given AM1710, and IL‐10 IR remained unchanged at neuropathic levels in CCI neuropathic mice given i.p. AM1710. (j, k, l) Representative spectrally unmixed images at 20x magnification of IL‐10 fluorescent labeling (green) with DAPI nuclear stain (blue) in spinal cord dorsal horn. (m) Compared to sham controls, IL‐1β expression was increased in the dorsal horn spinal cord of CCI‐treated animals given i.p. vehicle of AM1710. Intraperitoneal AM1710 in CCI‐treated mice did not alter increases in IL‐1β IR. (n, o, p) Representative spectrally unmixed images at 20x magnification of IL‐1β fluorescent labeling (red) with DAPI nuclear stain (blue) in spinal cord dorsal horn. (q) Iba‐1 IR expression increased within the ipsilateral dorsal horn of the spinal cord following CCI manipulations compared to control sham treatment, irrespective of i.p. vehicle or AM1710. ***p < .0001, **p < .001, *p < .05 versus. WT‐CCI‐Vehicle. Data reflect mean ± *SEM*, *n* = 3 mice per group

We prioritized examining DRG because the route of AM1710 was peripheral, and thus, we predicted peripheral nervous tissue would reflect changes in cytokine levels as a consequence of AM1710 exposure. While bilateral IL‐10 IR in corresponding DRG was diminished in CCI mice given i.p. vehicle compared to sham‐operated mice, regardless of genotype (Figure 5a Figure S3c,d), treatment with i.p. AM1710 raised IL‐10 IR in the DRG bilaterally. These levels were similar to those found in sham‐operated mice (ANOVA, *F*_(8,26)_ = 20.80; *p* < .0001 ANOVA, *F*_(8,26)_ = 11.31; *p* < .0001, respectively, for ipsilateral and contralateral DRG). Representative immunofluorescence images of ipsilateral DRG from CB_1_R (+/+) sham mice with i.p. vehicle (Figure 5b) and CCI mice with i.p. vehicle (Figure 5c) or with i.p. AM1710 (Figure 5d) corresponding to data examined by microscopic analysis are shown.

Bilateral DRG levels of increased IL‐1β IR in neuropathic mice given i.p. vehicle were decreased in CCI‐operated mice following treatment with i.p. AM1710, with levels comparable to sham‐operated mice (ANOVA, *F*_(8,26)_ = 25.30; *p* < .0001 ANOVA, *F*_(8,26)_ = 11.08; *p* < .0001, respectively) (Figure 5e, Figure S3e,f). Representative fluorescence images of ipsilateral DRG from CB_1_R (+/+) sham mice with i.p. vehicle (Figure 5f), CCI mice with i.p. vehicle (Figure 5g), or with i.p. AM1710 (Figure 5h) corresponding to the quantitative analysis are shown.

In an effort to examine a comprehensive analysis of the anti‐inflammatory‐like effects of i.p. AM1710, L4‐L6 dorsal horn of the spinal cord was additionally examined. It is important to note that we cannot rule out the possibility that AM1710 can cross the blood–spinal barrier and produce similar actions. The data show that IL‐10 IR in both the ipsilateral and contralateral dorsal horn of the spinal cord was significantly diminished in CCI‐operated mice given i.p. vehicle (ANOVA, *F*_(8,26)_ = 6.211; *p* = .0006 ANOVA, *F*_(8,26)_ = 6.810; *p* = .0004, respectively) (Figure 5i, Figure S3g,h). However, treatment with i.p. AM1710 did not reliably elevate spinal IL‐10 IR in CCI‐operated mice. Representative fluorescence images corresponding to data examined by microscopic analysis of ipsilateral spinal cord dorsal horn from CB_1_R (+/+) sham mice with i.p. vehicle (Figure 5j) or CCI mice with i.t. vehicle (Figure 5k) or with i.p. AM1710 (Figure 5l) are shown.

Upon examination of 1L‐1β IR within the dorsal horn spinal cord, CCI‐operated mice with neuropathy revealed a bilateral increase in 1L‐1β in i.p. vehicle‐injected animals (ANOVA, *F*_(8,26)_ = 145.2; *p* < .0001 ANOVA, *F*_(8,26)_ = 172.3; *p* < .0001, respectively) (Figure 5m, Figure S3i,j). Surprisingly, i.p. administration of AM1710 did not alter dorsal horn IL‐1β IR in CCI‐operated mice which displayed behavioral reversal of allodynia. Representative fluorescence images of ipsilateral spinal cord dorsal horn from CB_1_R (+/+) sham mice with i.p. vehicle (Figure 5n) or CCI mice with i.t. vehicle (Figure 5o) or with i.p. AM1710 (Figure 5p) corresponding to the quantitative data are shown.

Given the robust reversal from allodynia and the significant IL‐10 and IL‐1β IR changes found in the DRG following i.p. AM1710, possible indirect actions of AM1710 in the spinal cord may support the anti‐allodynic actions observed in these mice. That is, a reduction in the pronociceptive drive from DRG nerve terminals projecting to the spinal cord could reduce microglial activation and thereby reduce allodynia. Iba‐1 is a marker for microglial activity, and the data reveal that elevated Iba‐1 IR within the dorsal horn was observed in CCI‐operated mice given i.p. vehicle, and CCI mice treated with i.p. AM1710 revealed no changes in Iba‐1 IR compared to CCI mice with ongoing allodynia (ANOVA, *F*_(8,26)_ = 15.37; *p* < .0001 ANOVA, *F*_(8,26)_ = 8.777; *p* = .0001, respectively) (Figure 5q, Figure S3k,l). CCI surgery produced increased dorsal horn GFAP IR levels, a marker for altered astrocyte activity, and despite behavioral reversal, i.p. AM1710 did not alter GFAP IR in CCI‐treated mice (data not shown).

### 3.6 Immunohistochemical analysis of MCP‐1 in DRG and dorsal horn spinal cord

**Figure 6:**
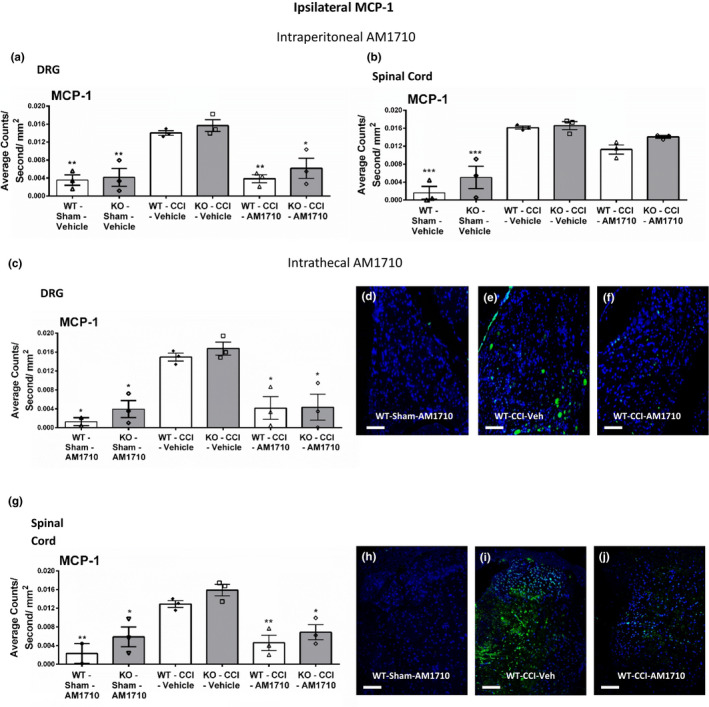
Ipsilateral immunofluorescent intensity quantification of MCP‐1. Ipsilateral immunofluorescent intensity quantification of DRG and spinal cord dorsal horn reveals both i.p. and i.t. AM1710 administration reduce the expression of the chemokine MCP‐1. (a, b) Compared to control mice, DRG and spinal cord dorsal horn MCP‐1 IR expression was increased in CCI‐treated neuropathic mice that received i.p. vehicle of AM1710, while DRG and spinal cord dorsal horn MCP‐1 IR in CCI‐treated mice given i.p. AM1710 was reduced. (c) Compared to control mice, DRG MCP‐1 IR expression was increased in CCI‐treated neuropathic mice that received i.t vehicle of AM1710, while DRG MCP‐1 IR in CCI‐treated mice given i.t. AM1710 was substantially reduced. (d, e, f) Representative spectrally unmixed images at 20x magnification of MCP‐1 fluorescent labeling (green) with DAPI nuclear stain (blue) in DRG. (g) Compared to control mice, spinal MCP‐1 IR expression was increased in CCI‐treated neuropathic mice that received i.t vehicle of AM1710, while spinal MCP‐1 IR in CCI‐treated mice given i.t. AM1710 was substantially reduced. (h, i, j) Representative spectrally unmixed images at 20x magnification of MCP‐1 fluorescent labeling (green) with DAPI nuclear stain (blue) in spinal cord dorsal horn. All sections were 7 μm in thickness. ***p < .0001, **p < .001, *p < .05 versus. WT‐CCI‐Vehicle. Data reflect mean ± *SEM*, *n* = 3 mice per group for all groups except *n* = 2 within the WT‐Sham‐i.t. AM1710 group

The well‐characterized chemotactic cytokine, MCP‐1, that is released from damaged DRG neurons and surrounding satellite DRG cells, was examined to determine whether AM1710 altered MCP‐1 IR expression levels. The data show that bilateral DRG MCP‐1 IR was dramatically increased in CCI‐operated mice of all genotypes, compared to sham‐operated mice receiving i.p. vehicle (Figure 6a, Figure S4a,b). Meanwhile in CCI‐operated mice, i.p. AM1710 significantly decreased bilateral MCP‐1 IR, with levels similar to sham controls (ANOVA, *F*_(8,26)_ = 13.39; *p* < .0001 ANOVA, *F*_(8,26)_ = 7.566; *p* = .0006, respectively, for ipsilateral and contralateral DRG). While peripheral administration of AM1710 (i.p.) leads to profound suppression of MCP‐1 in the DRG, only a modest reduction of MCP‐1 IR in the spinal cord was observed (ANOVA, *F*_(8,26)_ = 27.94; *p* < .0001 ANOVA, *F*_(8,26)_ = 21.29; *p* < .0001, respectively) (Figure 6b, Figure S4c,d).

Similar to peripheral administration, i.t. AM1710 resulted in dramatically reduced MCP‐1 IR in the DRG (the ipsilateral and contralateral DRG) (ANOVA, *F*_(8,24)_ = 4.782; *p* = .0038 ANOVA, *F*_(8,24)_ = 7.205; *p* = .0004; Figure 6c,d–f Figure S4e,f). Intrathecal AM1710 also substantially suppressed MCP‐1 IR within both the ipsilateral and contralateral spinal cord dorsal horn of CCI‐operated mice (ANOVA, *F*_(8,24)_ = 10.17; *p* < .0001 ANOVA, *F*_(8,24)_ = 6.371; *p* = .0009; Figure 6g,h–j, Figure S4g,h).

### 3.7 AM1710 reduces lipopolysaccharide effects in RAW264.7 cells

**Figure 7:**
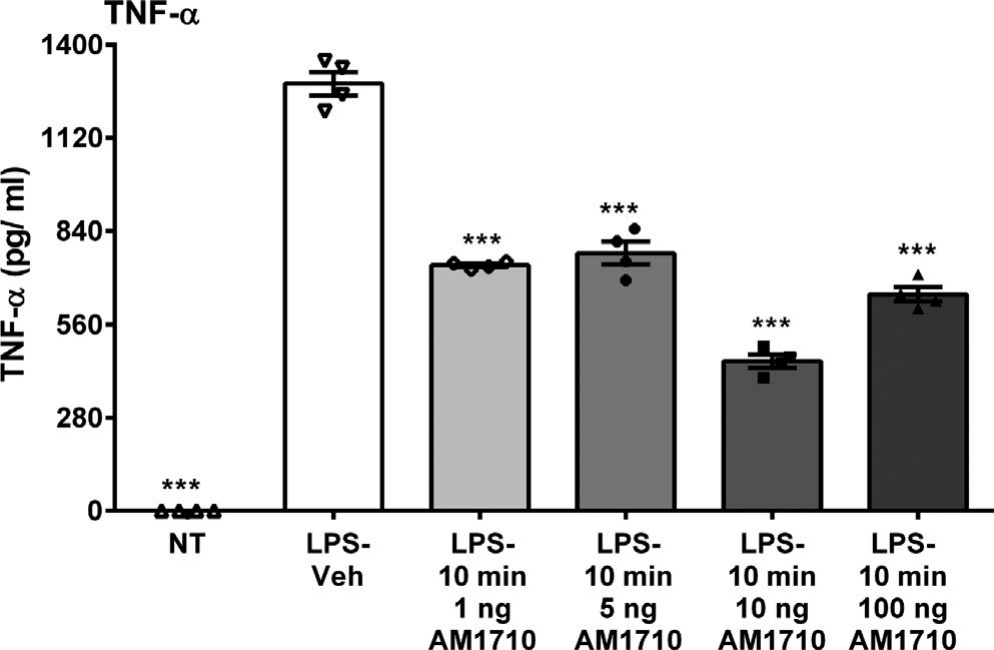
AM1710 reduces lipopolysaccharide effects in RAW264.7 cells. AM1710 reduces lipopolysaccharide effects in RAW264.7 cells. (a) LPS stimulation of RAW264.7 cells produced robust increases in TNF‐α protein. Treatment with AM1710 after LPS stimulation diminished TNF‐α protein levels

Compared to vehicle controls, LPS stimulation produced a robust increase in TNF‐α protein released into the supernatant of cultured RAW264.7 cells (ANOVA, *F*_(6,23)_ = 22.54; *p* < .0001) (Figure 7a). Treatment with varying doses of AM1710 significantly reduced TNF‐α protein levels irrespective of the doses applied that ranged from 1 to 100 ng.

## 4 DISCUSSION

Reduced copy number of CB_1_R did not alter allodynia under neuropathic conditions. These data support an anti‐inflammatory mechanism of AM1710 that is selective for the CB_2_R and is not reliant on actions of CB_1_R. Perispinal AM1710 produced changes in the IR of IL‐10, IL‐1β, and MCP‐1 in both spinal cord and DRG, with corresponding relief from allodynia. Interestingly, IL‐1β IR in the spinal cord appears to occur in white matter. While speculative, it is possible that within the white matter, a mixture of both infiltrating macrophage and resident microglia as well as reactive immature radial glia may express IL‐1β. With the reversal of allodynia following peripheral administration of AM1710, a compound that does not readily transverse the blood–brain barrier (Rahn et al., 2011), there were peripheral decreases of DRG MCP‐1 and cytokine IR. However, within the dorsal horn of the spinal cord at 30 min, there was no change in spinal IL‐1β or IL‐10 following peripheral AM1710. Interestingly, there was a discernible decrease in MCP‐1, indicating that spinal MCP‐1, and not IL‐10 or IL‐1β, may be a critical early modulator in the mechanisms of neuropathic pain reversal. In a cell culture model of macrophage stimulation, AM1710 decreased the proinflammatory cytokine TNF‐α.

### 4.1 Specificity of AM1710’s behavioral and cytokine effects

In support of work in rats (Wilkerson et al., 2012a), intrathecal AM1710 reversed mechanical allodynia as well as reset both spinal and DRG levels of IL‐10 and IL‐1β IR to basal levels in neuropathic mice in the presence of behavioral reversal, independent of CB_1_R. Intrathecal pretreatment with AM630, a CB_2_R antagonist, in CB_1_R (−/−) mice with CCI blocked the effects of AM1710. However, inclusion of wild‐type mice receiving i.t. AM630 would strengthen the interpretation of the specific effects of AM630. It is important to point out that prior reports have demonstrated a lack of effect of i.t. AM630 on sensory thresholds in vivo (Deng et al., 2015) or in vitro on spinal cord slices (Yang et al., 2016). Together, these data provide in vivo evidence that AM1710 acts on CB_2_R to suppress allodynia.

Few publications have looked at DRG changes with an intrathecal injection of preclinical compounds in development for the treatment of neuropathic pain (Wilkerson et al., 2012a, 2012b). Previously, we demonstrated that the Nuance spectral analysis is more sensitive than standard ImageJ analysis and demonstrated the ability to capture significant differences in IL‐1β due to CCI (Wilkerson et al., 2012b). We also previously found that under neuropathic pain conditions in rats, AM1710 increased DRG and spinal expression of IL‐10 and decreased the expression of IL‐1β as well as phospohorylated‐p38MAPK, and in the same report, we identified a reduction in TNF‐α protein content in DRG as measured by ELISA (Wilkerson et al., 2012a). Here, we report anatomically distinct protein profiles for IL‐10, IL‐1β, and MCP‐1 under conditions of neuropathy, which has not been widely examined. Both spinal cord and DRG IR are altered with acute i.t. AM1710 injection. However, following i.p. injection only, DRG at a 30‐min time point, reveal IL‐10 and IL‐1β IR changes that correspond to allodynia reversal. It may be under these conditions that an existing and dynamic neuron to glial component is important in mediating acute actions.

We examined the ability of peripheral CB_2_R to reverse allodynia by administering systemic AM1710 in CB_1_R (−/−) mice. We found that peripheral CB_1_R was dispensable in the anti‐allodynic actions of intraperitoneal AM1710. These findings expand upon previous reports, showing that AM1710’s peripheral anti‐allodynic effects in a chemotherapy‐induced peripheral neuropathy mouse model are not due to CB_1_R‐related mechanisms (Deng et al., 2012, 2015). These data support prior findings showing AM1710 does not effectively transverse the blood–brain barrier, and effects of i.p. AM1710 are faster than i.t. AM1710 (<30 min, versus. 90 min after administration, respectively) (Rahn et al., 2011; Wilkerson et al., 2012).

These data do not exclude the possibility that CB_2_R may be found on peripheral nerve terminals, especially under neuropathic conditions (Kress & Kuner, 2009). Indeed, peripheral AM1710 may act at either nociceptive terminals and/or neuronal cell bodies within the DRG. In support of peripherally restricted actions of systemically delivered AM1710, we show robust effects in the DRG of both IL‐10 and IL‐1β IR, as well as reduced IL‐10. Increases in IL‐1β IR, glial GFAP (data not shown), and Iba‐1 IR persisted in the spinal cord during acute allodynia reversal, which suggests that spinal mechanisms involving cytokines are not required to drive acute neuropathic pain. Short‐term actions (i.e., less than an hour) at the level of the DRG may lead to anti‐allodynia. However, these actions may terminate prior to IL‐10 effects, which might be seen with longer‐duration pain reversal.

### 4.2 Importance of MCP‐1 during neuropathic pain

The chemokine MCP‐1 is known to be released by neurons found within the dorsal horn of the spinal cord and is bound by its receptor, CCR2, found on immune cells (macrophage as well as other myeloid‐derived cells), and resulting CCR2 signaling cascades are characterized to attract monocyte‐derived cells to areas of tissue injury (Deshmane et al., 2009). These data support prior reports demonstrating increased expression of MCP‐1 in both spinal cord nociceptive neurons and interneurons, astrocytes, and DRG nociceptive neuronal cell bodies with CCI‐induced neuropathy (Gao et al., 2009; Gosselin et al., 2005; Jung et al., 2009; Tanaka, Minami, Nakagawa, & Satoh, 2004; Thacker et al., 2009; White et al., 2005; Zhang et al., 2007). However, we have extended these findings and show dynamic regulation of MCP‐1 with either i.p. or i.t. administration of the anti‐inflammatory CB_2_R agonist AM1710. Intrathecal AM1710 produced a significant decrease of MCP‐1 in both the spinal cord and DRG. Intraperitoneal AM1710 led to decreases in not only DRG MCP‐1 IR, but also in dorsal horn spinal cord MCP‐1 IR. This early alteration of spinal MCP‐1 after peripheral administration of AM1710 suggests that this chemokine may be a key neuronal driving factor regulating neuropathic pain.

Under pathological conditions, it was historically accepted that the actions of CCR2, due to MCP‐1 binding on resident spinal microglia, allowed for behavioral alterations to develop after peripheral nerve ligation (Zhang et al., 2007). However, the technological development of single‐cell RNA profiling has changed our understanding of CCR2 expression by resident CNS microglia. Relevant to this study, compared to mouse resident microglia, mouse infiltrating myeloid lineage cells exhibit ~127‐fold higher RNA expression of CCR2 (Zhang et al., 2014). Thus, it is controversial that resident CNS microglia express CCR2. It is likely that under pathological conditions, nonresident monocytes and macrophage infiltrate into the CNS and respond to aberrant MCP‐1 (Li & Barres, 2018). Indeed, pharmacological suppression of MCP‐1 under neuropathic conditions blocks the development of pain (Old & Malcangio, 2011). Additionally, overexpression of MCP‐1 is capable of producing allodynia in otherwise naïve animals (Menetski et al., 2007). It has been shown that release of MCP‐1 leads directly to the upregulation of IL‐1β (Old & Malcangio, 2011). One critical factor in controlling neuropathic pain may be increased by MCP‐1 actions. Importantly, CB_2_R agonists may participate in regulating MCP‐1‐mediated pain.

Here, we show that reversal of CCI‐induced allodynia is achieved after both intrathecal and intraperitoneal injection of the CB_2_R agonist AM170 and that CB_1_R actions are dispensable. Further, we demonstrate potentially distinct mechanistic effects on inflammatory mediator expression in the spinal cord when AM1710 is administered peripherally (i.p.) versus centrally (i.t.), despite a similar magnitude of antinociceptive effectiveness, albeit at different timepoints after injection. Notably, intraperitoneal administration of AM1710 led to a more expedient reversal of allodynia. We propose that changes at the sciatic nerve constriction site and corresponding DRG occur within minutes after injection, and this action at the DRG is reflected in our immunohistochemical data obtained at 30 min after intraperitoneal administration. Further, reduced neuronal drive to spinal cord is sufficient to reduce pain neuron excitability within the spinal cord (Woolf, 2004). However, with this transient acute behavioral reversal, changes in spinal IL‐10 or IL‐1β are not observed. Conversely, in order to observe behavioral reversal after an intrathecal injection with a CB_2_R agonist, it may be that first, a “winding‐down” of glia within the spinal cord is needed, which would then lead to decreases in neuronal excitability and subsequent reversal of observed allodynic behaviors. This study supports the development of cannabinoid agonists as a promising avenue for novel therapeutics for the treatment of pathological pain.

## 5 CONCLUSIONS

Here, in a mouse model of neuropathy, we have shown that CB_1_R is dispensable for both peripheral and central anti‐allodynic actions of AM1710. Additionally, intraperitoneal administration of AM1710 led to more expedient reversal of allodynia than intrathecal administration. We propose that anti‐inflammatory changes at the site of the damaged sciatic nerve and corresponding DRGs occur within minutes after injection. After an intrathecal injection with a CB_2_R agonist, glia within the spinal cord may require a reduction in proinflammatory tone prior to leading to decreases in neuronal pronociceptive drive resulting in reversal of observed allodynic behaviors. This study supports the development of cannabinoid CB_2_R‐preferring agonists as a promising avenue for novel therapeutics for the treatment of pathological pain.

## CONFLICT OF INTEREST

The authors declare that they have no conflict of interest.

## AUTHOR CONTRIBUTIONS

Wilkerson and Milligan participated in research design. Wilkerson, Alberti, and Kerwin conducted the experiments. Ledent, Thakur, and Makriyannis contributed to new reagents/analytic tools. Wilkerson and Milligan performed data analysis and wrote or contributed to the writing of the manuscript.

### Peer Review

The peer review history for this article is available at https://publons.com/publon/10.1002/brb3.1850.

## Supporting information

Fig S1‐S4

## Data Availability

The data that support the findings of this study are openly available at Open Science Framework at https://osf.io[DOI10.17605/OSF.IO/NV9FZ].
