# Supplementary material for: Peripheral versus central mechanisms of the cannabinoid type 2 receptor agonist AM1710 in a mouse model of neuropathic pain
Source: Brain Behav. 2020 Sep 25;10(12):e01850. doi: 10.1002/brb3.1850 (PMC7749576; doi:10.1002/brb3.1850)

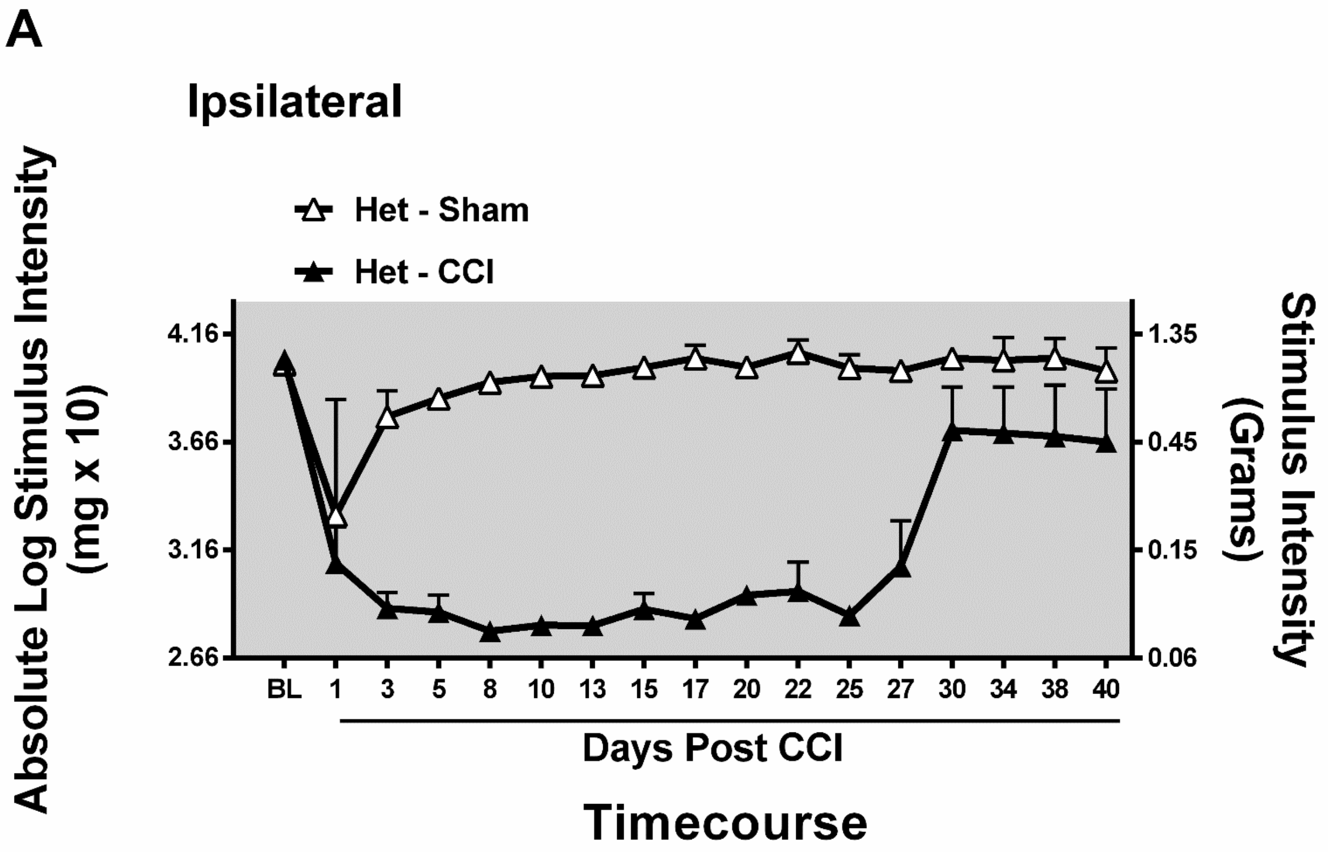
A
Ipsilateral
Het - Sham
Het - CCI
Absolute Log Stimulus Intensity (mg x 10)
4.16
3.66
3.16
2.66
Stimulus Intensity (Grams)
1.35
0.45
0.15
0.06
BL
1
3
5
8
10
13
15
17
20
22
25
27
30
34
38
40
Days Post CCI
Timecourse

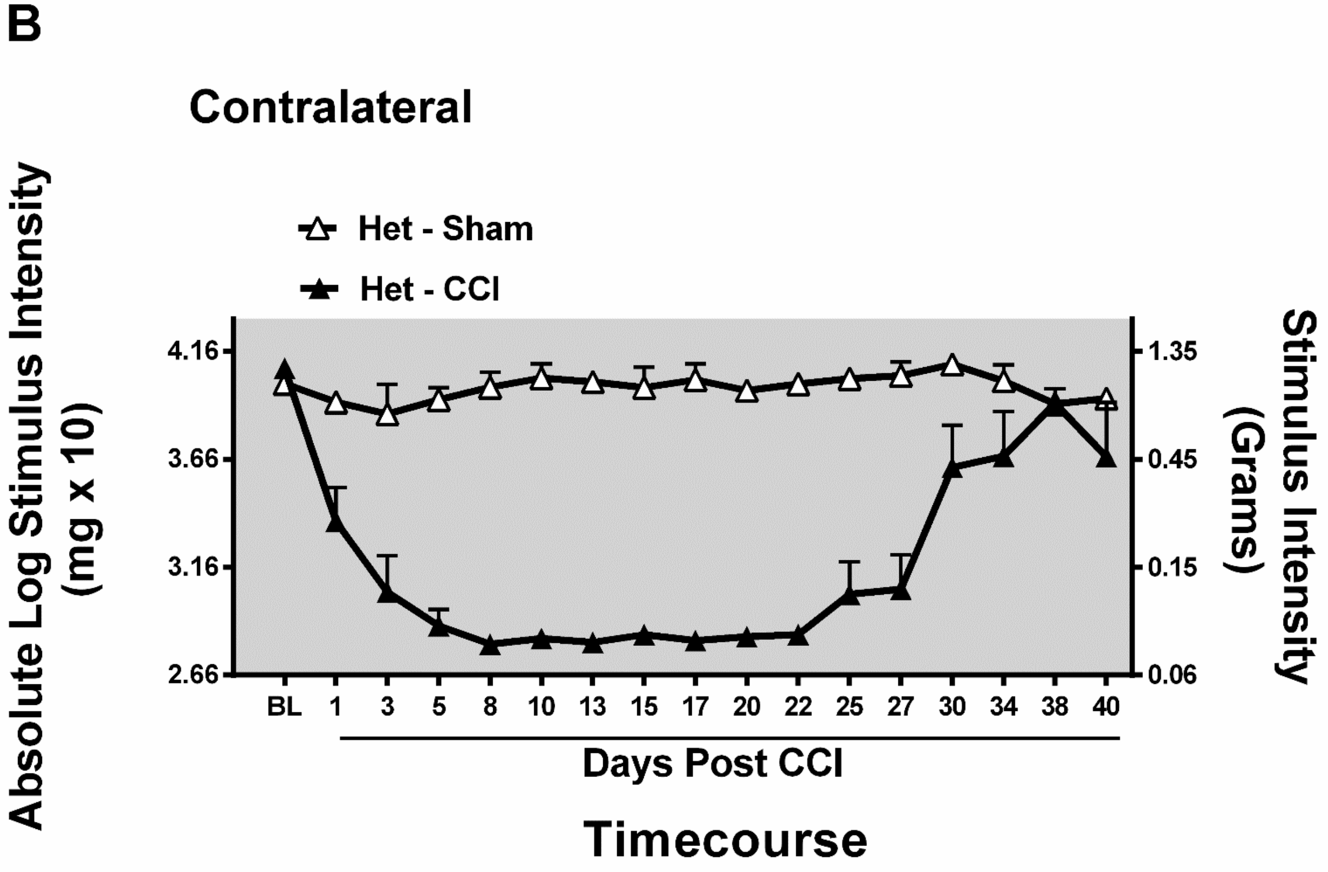
B
Contralateral
Het - Sham
Het - CCI
Absolute Log Stimulus Intensity (mg x 10)
4.16
3.66
3.16
2.66
Stimulus Intensity (Grams)
1.35
0.45
0.15
0.06
BL
1
3
5
8
10
13
15
17
20
22
25
27
30
34
38
40
Days Post CCI
Timecourse

Supplemental Figure 2 Wilkerson et al.

Intrathecal AM1710

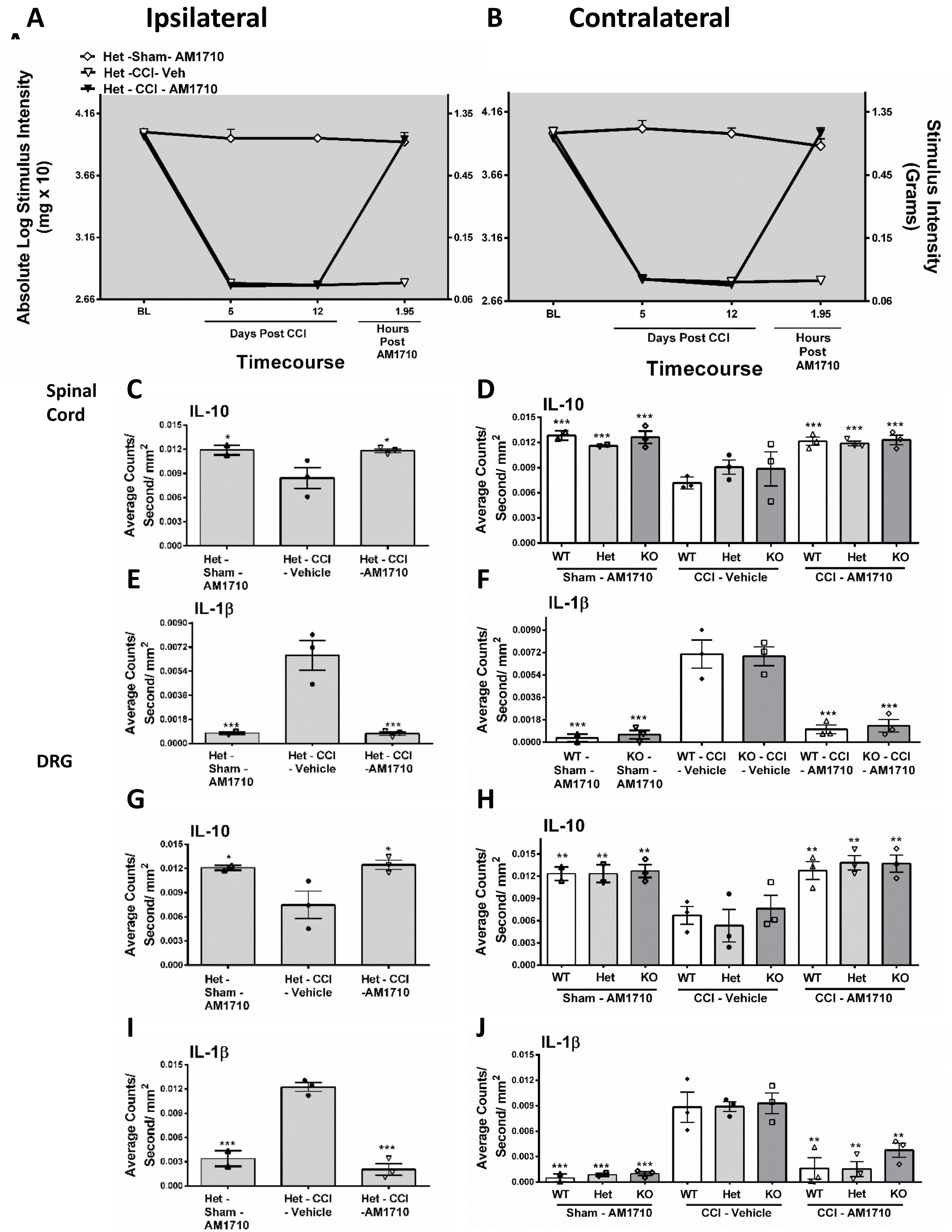

Supplemental Figure 3

Intraperitoneal AM1710

Wilkerson et al.

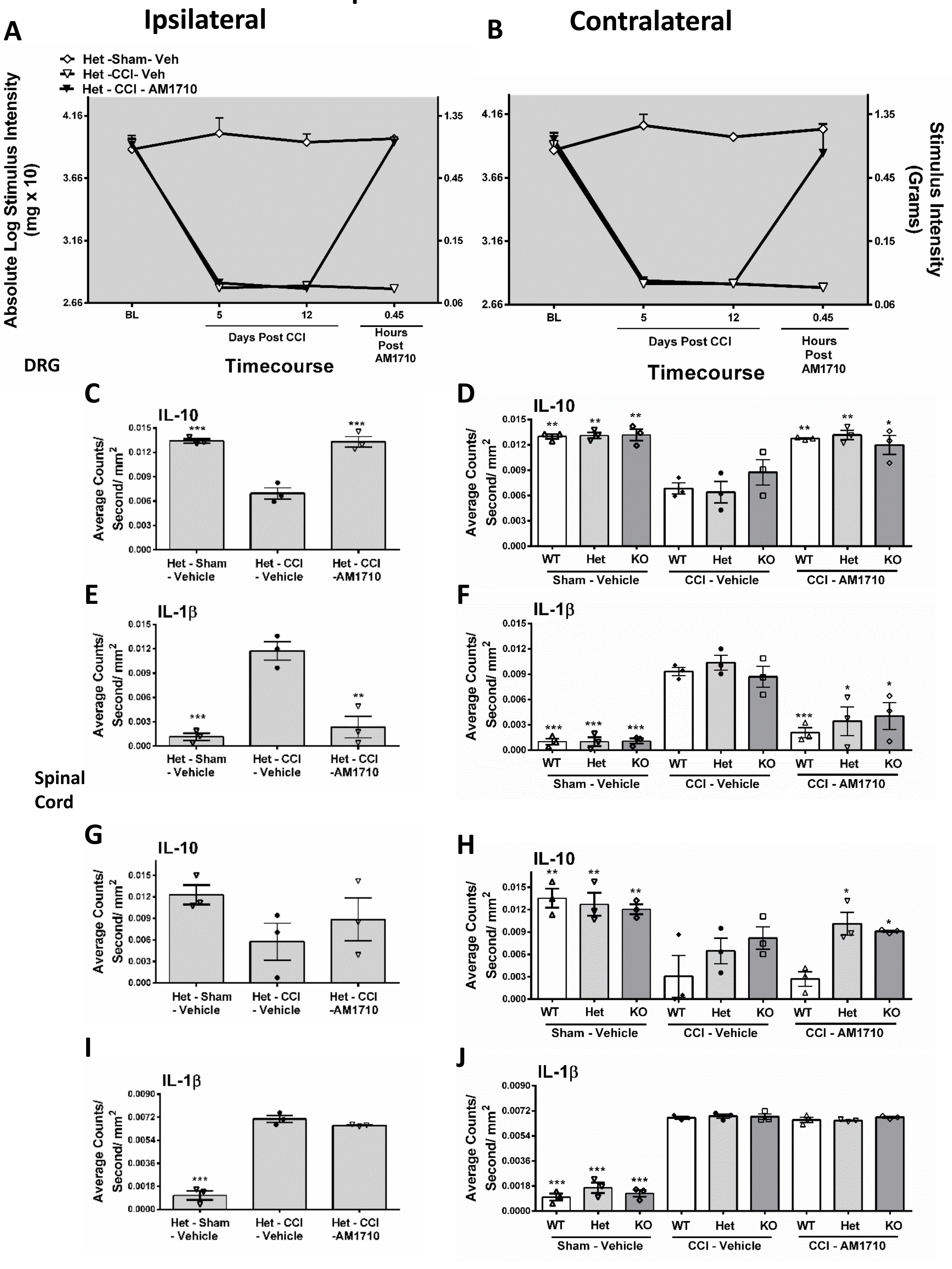

Supplemental Figure 3

Intraperitoneal AM1710

Wilkerson et al.

K

Ipsilateral

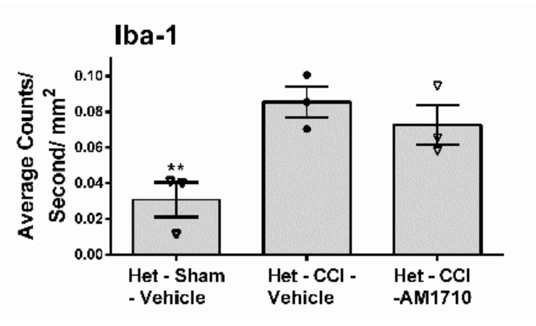

L

Contralateral

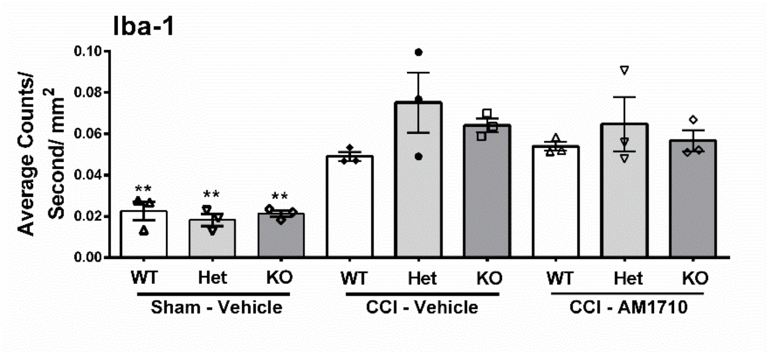

Supplemental Figure 4 Wilkerson et al.

Ipsilateral **Contralateral**

Intraperitoneal AM1710

DRG

A

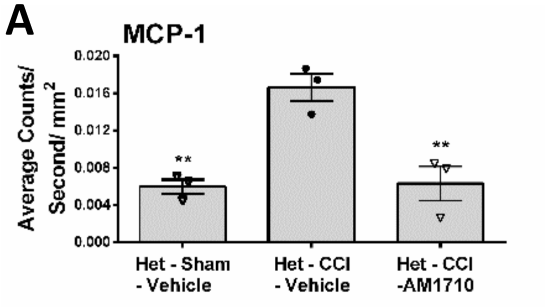

B

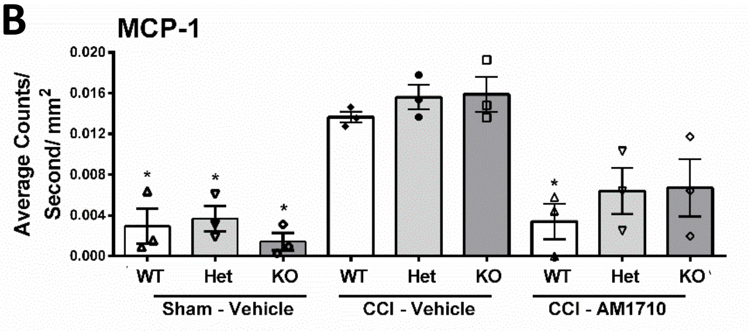

Spinal Cord

C

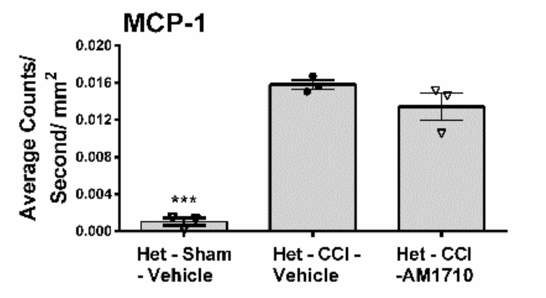

D

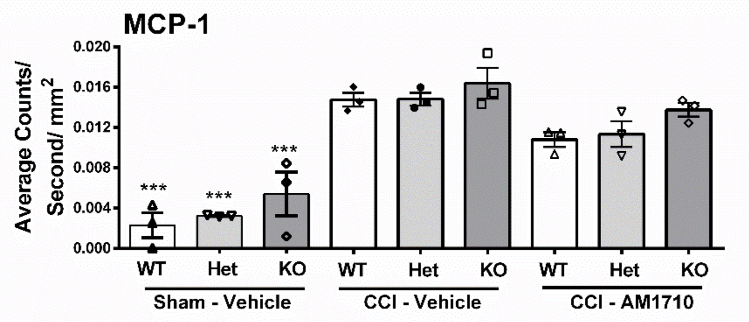

Intrathecal AM1710

DRG

E

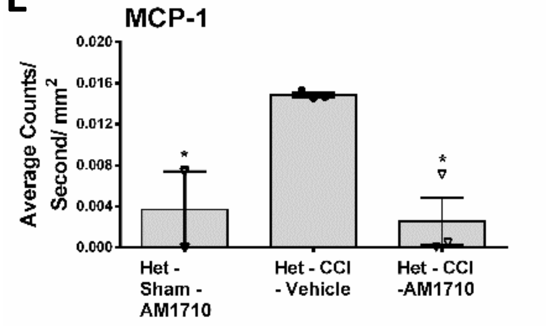

F

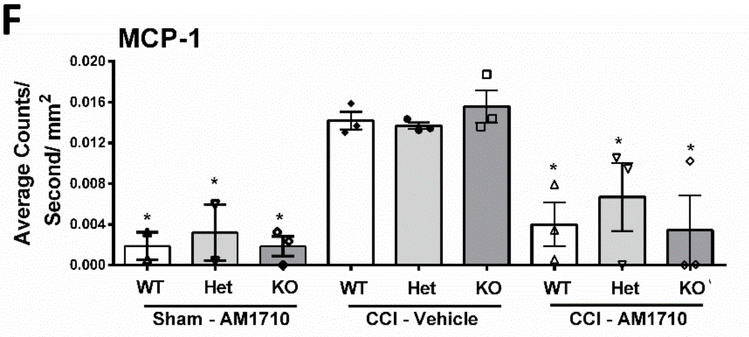

Spinal Cord

G

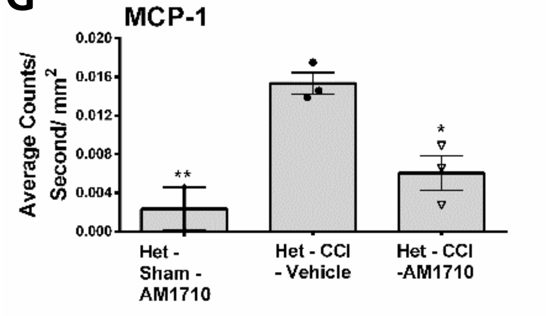

H

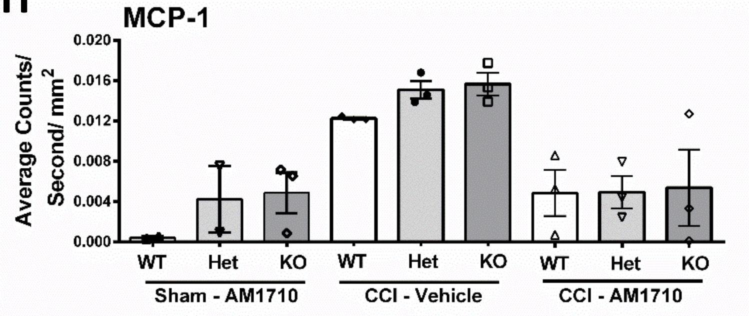

Supplement: Supplementary file 1 — Fig S1‐S4 [file BRB3-10-e01850-s001.pdf]
